# Payment in challenge studies: ethics, attitudes and a new payment for risk model

**DOI:** 10.1136/medethics-2020-106438

**Published:** 2020-09-25

**Authors:** Olivia Grimwade, Julian Savulescu, Alberto Giubilini, Justin Oakley, Joshua Osowicki, Andrew J Pollard, Anne-Marie Nussberger

**Affiliations:** 1 Faculty of Medicine, Nursing and Health Sciences, Monash University, Clayton, Victoria, Australia; 2 Oxford Uehiro Centre for Practical Ethics, University of Oxford, Oxford, UK; 3 Murdoch Childrens Research Institute, Parkville, Victoria, Australia; 4 Melbourne Law School, University of Melbourne, Melbourne, Victoria, Australia; 5 Wellcome Centre for Ethics and Humanities, University of Oxford, Oxford, UK; 6 Monash Bioethics Centre, Monash University, Melbourne, Victoria, Australia; 7 Tropical Diseases Research Group, Murdoch Childrens Research Institute, Parkville, Victoria, Australia; 8 Department of Paediatrics, The University of Melbourne, Melbourne, Victoria, Australia; 9 Department of Paediatrics, University of Oxford, Oxford, UK; 10 Department of Experimental Psychology, University of Oxford, Oxford, UK

**Keywords:** research ethics, coercion

## Abstract

Controlled Human Infection Model (CHIM) research involves the infection of otherwise healthy participants with disease often for the sake of vaccine development. The COVID-19 pandemic has emphasised the urgency of enhancing CHIM research capability and the importance of having clear ethical guidance for their conduct. The payment of CHIM participants is a controversial issue involving stakeholders across ethics, medicine and policymaking with allegations circulating suggesting exploitation, coercion and other violations of ethical principles. There are multiple approaches to payment: reimbursement, wage payment and unlimited payment. We introduce a new Payment for Risk Model, which involves paying for time, pain and inconvenience and for risk associated with participation. We give philosophical arguments based on utility, fairness and avoidance of exploitation to support this. We also examine a cross-section of the UK public and CHIM experts. We found that CHIM participants are currently paid variable amounts. A representative sample of the UK public believes CHIM participants should be paid approximately triple the UK minimum wage and should be paid for the risk they endure throughout participation. CHIM experts believe CHIM participants should be paid more than double the UK minimum wage but are divided on the payment for risk. The Payment for Risk Model allows risk and pain to be accounted for in payment and could be used to determine ethically justifiable payment for CHIM participants.

Although many research guidelines warn against paying large amounts or paying for risk, our empirical findings provide empirical support to the growing number of ethical arguments challenging this status quo. We close by suggesting two ways (value of statistical life or consistency with risk in other employment) by which payment for risk could be calculated.

## Background

Challenge Studies, more formally known as Controlled Human Infection Model (CHIM) research studies, involve the infection of otherwise healthy participants with disease. CHIMs are employed in medical research for varied reasons, primarily to study causation of disease, incubation periods, clinical symptomology and most importantly to advance drug and vaccine development.^1^ Compared with traditional field trials, in which a novel vaccine is given to a large sample group and the incidence of disease is consequently assessed, early vaccine evaluation using CHIMs offer numerous advantages; in particular, they are cost and time effective because they allow for the quick differentiation between promising and poor vaccine candidates. This allows the development of effective vaccines to be accelerated, while poor vaccines can be discarded to prevent further expensive, wasteful and unsuccessful trials.^2^ CHIMs also require only a small number of participants, which means that fewer people are subjected to the risks involved with taking a novel vaccine.^1^ Partly for these reasons, CHIM research has recently experienced a resurgence in popularity with an estimated 22 000 participants involved in CHIMs over the past 70 years.^3^ Recent CHIMs have led to many clinically valuable breakthroughs including the proof of efficacy of the new oral cholera vaccine, Vaxchora (CVD 103-HgR),^4^ and the proof of efficacy for a Vi-tetanus toxoid conjugate vaccine.^5^

The current COVID-19 pandemic has led to millions of cases and hundreds of thousands of deaths. It underscores the importance of supporting and fostering effective fast track pathways for testing vaccines. Quickly discovering a vaccine for the SARS-CoV-2 responsible for COVID-19 will save millions of lives. To this end, it is unsurprising a coronavirus CHIM using less virulent strains is already in the pipeline,^6^ and there are increasing calls for a SARS-CoV-2 CHIM.^7^ Both of which are likely to have prompted the WHO to release an ethical guidance document for these studies.^8^ Growing antimicrobial resistance^9^ and concerns of climate change increasing the climate suitability for the transmission of infectious diseases^10^ are further issues highlighting the pressing need for more CHIMs.

There has been much debate surrounding the ethical issues that CHIMs raise, namely regarding informed consent, acceptable levels of risk in research and the payment of participants.^11 12^ The intentional introduction of disease to participants by medical professionals may seem to contradict the Hippocratic principle of ‘first do no harm’. Unease surrounding CHIMs may also arise from unethical studies performed in the past on vulnerable populations without consent. For instance, in the 1960s, children with intellectual disabilities at The Willowbrook State School in New York were purposely infected with hepatitis.^13^ Despite this dark past, there is a strong argument for the continued support of this research, as long as it is properly regulated through appropriate Institutional Review Board (IRB) oversight, due to its potential to prevent suffering and deaths from infectious disease.^12^ It is important to note that central to any CHIM is a focus on participant risk minimisation through rigorous participant screening, often using an inpatient setting and employing altered, safer strains of treatable pathogens.^2^ Thanks to these safety measures, only four participants have ever experienced serious adverse events in modern CHIMs,^1^ a rate that is comparable with that reported in phase I drug trials.^14^

The payment of research subjects in CHIM warrants more discussion than has so far been undertaken. At the centre of the debate about payment lies the fear that the offer of money could be either coercive or an ‘undue inducement’ to research subjects.^15^ Currently, there is no clear consensus regarding what makes a payment coercive or an undue inducement or what these terms exactly mean. Furthermore, there is debate about whether these concerns are only theoretical or whether they exist in practice and hence warrant preventative action by researchers limiting participants’ payments.^16 17^ Whether there is ethical justification for explicitly accounting for the risk involved in a CHIM into the payment is also hotly disputed.^11 12 18^

At the moment, many published CHIMs do not clearly state what payment participants in their study received, and there has been no research into the payment practices across different models. Specific CHIM guidelines either do not mention payment at all,^8 19^ or despite recognising payment to be a ‘particularly sensitive issue’, they refer to more general medical research guidelines that offer non-specific advice reflective of the ongoing ethical debate surrounding appropriate payment for participants.^15^ Many general guidelines warn against paying for the risk in medical research (some even going as far as to accuse it of being ‘ethically unacceptable’) and against offering large payments for participation in medical trials on the basis of concerns about coercion and/or undue inducement.^20–22^ Moreover, without data detailing the actual payment processes in CHIM, some of the growing concerns about payments in medical research might be applicable to CHIMs. In particular, concerns have been voiced that levels of payment may vary considerably across studies and that many studies may be at risk of exploiting participants by underpaying them and providing improper compensation for the burdens and risk they bear through participation.^17 23–25^ As a consequence, there have been calls for higher, unrestricted payment in CHIMs that explicitly takes into account the risk involved in a specific trial.^18^

To date, there have been no studies that have assessed attitudes towards payment in CHIM either among the general public or CHIM experts. Although stakeholder attitudes cannot be used to deliver normative claims alone, when they are considered alongside pre-existing ethical theory, they can meaningfully contribute to both ethical reflection and policy (a procedure we have described elsewhere).^26^ The aim here is to establish coherence between attitudes and ethical arguments that can then inform the foundation of ethical policy. Level of payment should be set by the same process, which will include consistency with existing labour laws. CHIM experts are involved in proposing payment plans and are the target audience for any proposed ethical payment framework. Additionally, considering that CHIM research has the potential to immensely benefit society, maintaining the public’s trust in this research through identifying and addressing their opinions is paramount. Losing the public’s confidence could significantly impede or even completely stop this research. Mainstream media reporting on payment in different CHIMs, especially on the payment in COVID-19 CHIMs, has prompted increased public attention and ethical discussion on this issue that further emphasises the urgent need for a clear ethical framework to guide payments in CHIMs.^27–33^ Our study aimed to: (1) provide empirical data surrounding current payment practices in CHIMs and the attitudes towards payment in CHIMs; (2) critically assess these data to determine whether current practices and attitudes are ethically justified; and (3) propose a framework for ethically justifiable payment of CHIM participants.

## Methods

### Sample

We undertook a cross-sectional study in the form of two online surveys of two distinct sample groups: CHIM experts and UK citizens. CHIM experts were recruited via email in conjunction with the registration to the International Alliance for Biological Standardisation (IABS) – 3rd Human Challenge Trials in Vaccine Development Conference, which occurred in Oxford, UK, in February 2020 and brought together scientific, regulatory and ethical experts involved in CHIM research. These respondents were unpaid and older than 18 years. A representative sample of the UK public, representative in terms of gender, age and ethnicity distribution, was recruited through the online research platform Prolific Academic (PA). PA provides many researchers with valuable access to a large pool of research subjects and facilitates survey respondent recruitment in exchange for payment.^34^ Eligible PA respondents were UK citizens aged over 18 years. We aimed to recruit a minimum of 300 respondents, and each was paid £9/hour for completion of the survey (higher than the UK minimum wage).

### Survey

The two sample groups answered slightly different surveys, with CHIM experts answering a shortened survey due to time constraints. The surveys were developed with experts in experimental psychology, CHIM and bioethics to assess the content validity of the questions. Furthermore, both surveys were pretested by a group of medical students, and the public survey was piloted formally through PA for face validity.

#### Payment attitudes: experts and public

The survey concurrently made use of hypothetical vignettes and direct attitudinal questioning to thoroughly assess respondents’ attitudes towards payment in CHIMs. Hypothetical vignettes are useful tools commonly employed in empirical bioethics research to ascertain respondents’ attitudes in a realistic, specific and depersonalised context.^35 36^ The vignette block aimed to assess attitudes towards payment for risk and hence the independent variable was the level of risk involved in the hypothetical CHIM. Four different levels of risk (1 in 1 million, 1 in 100 000, 1 in 10 000 and 1 in 1000) were assessed across two different risk categories: the risk of ‘a serious side effect’ and the risk of ‘death from a serious side effect’^1^. Respondents were asked to indicate on a slider scale their willingness to allow participation (WTAP) in the given CHIM and what they believed should be the required payment for participation in the CHIM. As a reference point in the payment questions, respondents were provided with the approximate UK minimum wage. The order in which the two risk categories was presented to the respondents was counterbalanced in the survey by randomising respondents to two groups with each group receiving a different order of presentation of the two risk categories. The direct attitudinal questions asked respondents to rank the importance of different payment factors and to indicate their level of agreement with some statements regarding payment. Finally, respondents answered questions on basic demographic data (age, gender, level of education and income).

#### Payment practices: experts only

CHIM experts were asked to identify one CHIM they have been involved in and provide data about the nature of the study (the pathogen used, location and number of participants), the patient experience (time requirements, disease symptoms and investigations required), the risk involved (risk and occurrence of complications), the payment (currency, amount and factors considered in payment) and finally whether they believe the payment was appropriate.

#### Additional payment attitudes questions: public only

The public respondents answered two additional vignette blocks and some further direct attitudinal questions. These questions mainly explored the publics’ perceptions of different payment models and their attitudes towards coercion and undue inducement. The detailed description of these questions and their results have been reported in the supplementary material. Public respondents also completed the Falk Risk Preferences Staircase Module, which is a time effective and experimentally validated measure of general risk preferences^37^ for which we controlled in our statistical analysis. Additionally, we included a comprehension check and two attention checks throughout the public survey and subsequently excluded those who failed these checks from the final statistical analysis.

## Results

### Respondents

We received 63 survey responses from CHIM experts following the email recruitment of those registered for the IABS conference. Following the exclusion of incomplete and duplicate responses, the final sample size was 36. From this sample, 25 respondents reported complete payment practices data for a CHIM they were involved in. A UK representative sample of 300 respondents was recruited on PA. Following the exclusion of incomplete and duplicate submissions the sample size was 298. Of these 34 (11.4%) failed at least one of the two attention checks resulting in a final sample of 264. All the PA respondents were UK citizens and prior to excluding invalid responses, the proportions of gender, age and ethnic group resembled those that exist in the UK. Our CHIM experts were multinational; however, over a third (36.1%) were British (see supplements for the detailed demographic analysis).

### Attitudes towards participation and payment: experts and public

We performed mixed effect linear regression models to explore our main dependent variables—WTAP and required payment—across levels of risk, types of risk and respondent groups. These models allowed us to account for the nested structure of the data and were implemented in *R* using the *lme4* package.^38^ Besides subject-level random intercepts, we defined *level of risk* (1 in 1 million, 1 in 100 000, 1 in 10 000 and 1 in 1000), *type of risk* (severe side effects vs death) and their interaction as fixed effects in our main models. We also ran models comparing across *respondent groups* (public vs experts). Additional models with controls for covariates such as gender, age, income and baseline risk preferences of public respondents were undertaken, and these variables did not affect our findings. Since our dependent variables were not normally distributed for either sample group (public WTAP: *W*=0.92, p<0.001, expert WTAP: W=0.87, p<0.001, public payment: *W*=0.88, p<0.001, expert payment: W=0.85, p<0.001), all analyses reported in the following were performed on natural log-transformed variables.

#### Willingness to allow participation

##### Public

**Figure 1:**
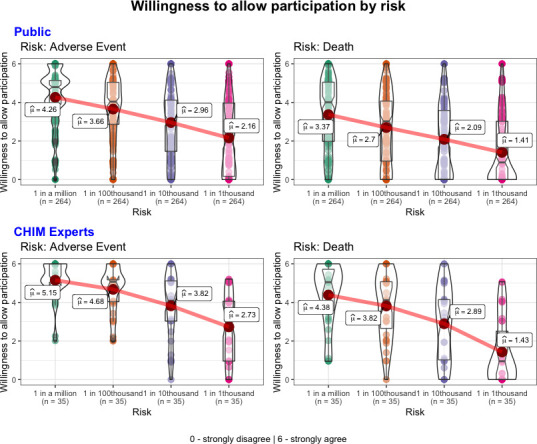
WTAP as a function of risk. Respondents indicated their WTAP (y-axis) on a slider scale from 0: strongly disagree to 6: strongly agree in response to the statement ‘*(the potential participant) should be allowed to participate in the study*’. This was indicated for each hypothetical study involving each risk category (x-axis). Mean values are labelled. CHIM, Controlled Human Infection Model; WTAP, willingness to allow participation.

We observed significant main effects for both type of risk (*χ* ^2^(1)=68.55, p<0.001) and level of risk (*χ* ^2^(3)=523.07, p<0.001), which were not qualified by an interaction (*χ* ^2^(3)=3.08, p=0.38). Thus, across all levels of risk, respondents’ WTAP was lower for risk of death compared with risk of adverse side effects (*b*=−0.25, p<0.001) and higher levels of risk (*b*s≥−0.16, p<0.001; see figure 1). These results replicated when we controlled for gender, age, income, belief about the scenario’s protagonist’s sex and baseline risk preferences.

##### Expert

We observed significant main effects for both type of risk (*χ* ^2^(1)=4.89, p=0.03) and level of risk (*χ* ^2^(3)=76.87, p<0.001); that were qualified by an interaction (*χ* ^2^(3)=8.66, *p*=0.03).Across all levels of risk, respondents’ WTAP was lower for risk of death compared with risk of adverse side effects (*b*=−0.18, *p*=0.03). Experts’ WTAP was lower for higher levels of risk (*b*s≥−0.10, p>0.05) and this pattern was pronounced for risk of death (see figure 1). These results replicated when we controlled for gender, age and income.

#### Required payment

##### Public

**Figure 2:**
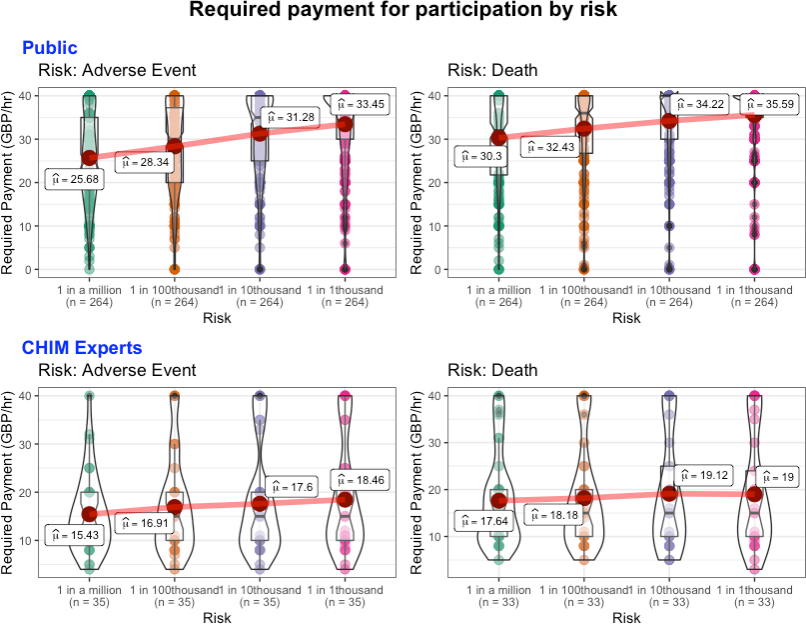
Required payment as a function of risk. Respondents indicated required payment in £/hour on slider scale from £0 to >£40 (y-axis) in response to each risk category (x-axis). An approximate minimum wage in the UK of £8/hour was provided to respondents as a reference point. Mean values are labelled. CHIM, Controlled Human Infection Model.

We observed significant main effects for both type of risk (*χ* ^2^(1)=23.64, p<0.001) and level of risk (*χ* ^2^(3)=75.91, p<0.001), which were not qualified by an interaction (*χ* ^2^(3)=6.40, p=0.09). Thus, across all levels of risk, respondents’ required payment was higher for risk of death compared with risk of adverse side effects (*b*=0.15, p<0.001) and higher levels of risk (*b*s≥0.08, p<0.01; see figure 2). These results replicated when we controlled for gender, age, income, belief about the scenario’s protagonist’s sex and baseline risk preferences

##### Experts

We observed a significant main effect for type of risk (*χ* ^2^(1)=5.03, p=0.02), but the effect was not significant for level of risk (*χ* ^2^(3)=7.41, p>0.05); this was not qualified by an interaction (*χ* ^2^(3)=1.35, p=0.72). Thus, across all levels of risk, respondents’ required payment was higher for risk of death compared with risk of adverse side effects (*b*=0.11, p=0.03) and higher levels of risk (*b*s≥0.06, p>0.05; see figure 2). These results replicated when we controlled for gender, age and income.

#### Comparison of the public and experts’ responses

To compare how the public and experts responded differently, we added respondent group as a fixed effect to our model. We observed a significant main effect for respondent group when it came to respondent’s WTAP (*χ* ^2^(1)=30.44, p<0.001) and payment (*χ* ^2^(1)=42.14, p<0.001). Thus, across all levels of risk and over the two risk categories, experts’ WTAP was higher than public WTAP (*b*=0.32, p<0.001) and experts’ payment was lower than public payment (*b*=−0.41, p<0.001). These results replicated when we controlled for gender, age and income.

#### Explicit rankings of payment factors

Explicit rankings of payment factors differed significantly between the two groups (table 1). In particular, the public ranked risk as more important compared with experts. Additionally, a lower proportion of the public believed that risk should not be taken into account when determining CHIM payment as compared with experts (1.1% vs 37.1%).

**Table 1:** Payment factors

Payment factor	Mean rank of importance *Mean (SD*)			% of participants who thought factor should *not* be accounted for in payment	
	Public n=264	CHIM experts n=33	T-test, p value	Public n=264	CHIM experts n=35
Risk of serious side effects and death involved in the study	1.24 (0.80)	3.85 (2.45)	t(32.86)=−6.08, p<0.001	1.1	37.1
Pain involved in the study	2.48 (0.86)	4.00 (1.20)	t(36.25)=−7.06, p<0.001	0.8	8.6
Number of invasive investigations involved in the study (eg, blood tests and investigations requiring sedation)	3.18 (1.01)	2.67 (1.02)	t(40.32)=2.73, p=0.009	0.4	2.9
Time required in the study	4.29 (1.43)	2.48 (1.75)	t(37.61)=5.69, p<0.001	1.5	0
Number of non-invasive investigations involved in the study (eg, urine sample, saliva swab and ultrasound)	4.95 (1.00)	4.58 (1.25)	t(37.30)=1.67, p>0.05	7.6	8.6
Inconvenience involved in the study	4.97 (1.12)	3.55 (1.50)	t(36.62)=5.28, p<0.001	10.6	0

Public and expert respondents were asked to rank the list of payment factors in order of importance when determining payment for CHIM participants. They were also asked if any payment factor should not be accounted for in payment. The mean rank of importance for each payment factor was compared between CHIM experts and public respondents using t-tests.

CHIM, Controlled Human Infection Model.

#### Agreement with payment statements

The public indicated a much higher level of agreement with statements supporting risk payment and harm compensation as compared with experts (table 2). The public indicated a mean level of agreement higher than 3 (neutral) for both statements outlining concerns that payment could be coercive or an undue inducement (table 2).

**Table 2:** Agreement with payment statements

Mean level of agreement Mean (SD)			% respondents who indicated a level of agreement greater than 4 – ‘somewhat agree’		Concept	Statement describing payment attitude
Public n=264	CHIM experts n=36	T-test, p value	Public n=264	CHIM experts n=36		
5.30 (1.07)	2.47 (2.02)	t(37.72)=8.24, p<0.001	91.7	27.8	Risk payment and harm compensation	Risk should be accounted for in the payment and extra compensatory payment should be given for actual harm.
2.25 (1.95)	2.88 (2.24)	t(41.09)=−1.59, p=0.12	25	45.7	No risk payment but harm compensation	Risk should not be accounted for in the payment but extra compensatory payment should be given for actual harm.
3.65 (1.67)	NA	NA	57.2	NA	Payment as coercion	Offering large amounts of money to become a Challenge Study participant could force people to participate in this research.
4.21 (1.41)	NA	NA	71.6	NA	Payment as an undue inducement	Offering large amounts of money to participants for being involved in Challenge Studies could impair participants’ judgement. This means they may choose to become involved in the research despite it not being in their best interests.

Respondents were asked to indicate their level of agreement with each statement on a slider scale from 0: strongly disagree to 6: strongly agree. The mean level of agreement for each statement was compared between CHIM experts and public respondents by using t-tests. As the CHIM experts survey did not include all statements, ‘NA’ indicates the questions that were not in the experts’ survey.

CHIM, Controlled Human Infection Model.

#### Agreement with payment models

The Payment for Risk Model and the Market Model were the most preferable models of payment for CHIM participants as indicated by receiving the highest mean levels of agreement from public respondents (table 3).

**Table 3:** Agreement with payment models: public respondents only

Mean level of agreement Mean (SD) n=*264*	% respondents who indicated a level of agreement greater than 4 – ‘somewhat agree’	Payment model	Statement describing payment model
0.33 (0.67)	0.76	No payment	Research participants should never be paid or reimbursed for their involvement in a Challenge Study.
1.05 (1.09)	1.89	Reimbursement model	Research participants should be paid for their involvement in a Challenge Study. Payment should aim to only reimburse the participant for time and travel expenses.
1.09 (1.13)	4.55	Wage payment model	Research participants should be paid for their involvement in a Challenge Study. The hourly rate of payment should be determined by what other unskilled labourers are paid, but no extra money should be given for the risks involved.
3.48 (1.83)	55.30	Payment for risk model	Research participants should be paid for their involvement in a Challenge Study. The base hourly rate of payment should be determined by what other unskilled labourers are paid with extra money given dependent on the risks involved.
3.42 (1.61)	48.11	Market model	Research participants should be paid for their involvement in a Challenge Study. Payment should be determined by the research investigators and funders, and it should depend on the current market demands (the demand/supply of participants and how quickly recruitment needs to occur).

Public respondents were asked to indicate their level of agreement with each statement on a slider scale from 0: strongly disagree to 6: strongly agree. The payment models provided were based on those proposed by Dickert and Grady.^52^

### Payment practices: experts only

Payment data were reported for 25 CHIMs involving 17 different pathogens. There was substantial variation in both the nature and the payment of the CHIMs. Sixteen (64%) of CHIMs were conducted in a high-income country (HIC) and 9 (36%) were conducted in a low-income or middle-income country (LMIC). Almost all CHIM participants were required to undertake investigations (92%), and over half of CHIM participants were expected to experience symptoms of disease (56%). The rates of payment varied considerably among CHIMs with the range of total payment varying from £0 (no payment) to £3393 (mean (SD)=1325.9 (1206.15)) and the range of hourly payment varying from £0 to £30 GBP (mean (SD)=10.51 (8.82)). Variation was also observed in similar CHIMs, with two CHIMs involving the same pathogen both taking place in an LMIC, paying inpatient daily rates of 15.36 GBP and 38.50 GBP, respectively.

Eighteen experts provided information about the factors that were taken into account when determining payment for their study (table 4). Time requirements and payment in previous studies were the most commonly taken into account, and eight (44.4%) of CHIMs took risk into account. No expert believed payment was too high in their CHIM, 21 (84%) believed payment was appropriate and 4 (16%) believed that the payment was too low in their studies. The reasons given for payment being too low mentioned risk or invasive investigations not being accounted for in payment, regulatory bodies not allowing more than no/minimal payment and payment not accurately compensating for time. In most CHIMs, the study team were involved with deciding payment (72%), and in about a third of CHIMs, an Human Research Ethics Committee (HREC) was involved in deciding on payment (32%). In terms of the risk involved in the CHIMs, 20 (80%) reported that the pathogen used can be associated with severe reversible complications and 16 (64%) reported that the pathogen can be associated with fatal complications, although none of these complications occurred during any of the CHIMs.

**Table 4:** Factors taken into account in payment for CHIM participation: experts only

Payment factor	No. of CHIMs reported to take factor into account in payment (%) (n=18)
Time requirements for participant	15 (83.3)
Payment in previous studies	15 (83.3)
Inconvenience	13 (72.2)
Invasive investigations	11 (61.1)
Risk	8 (44.4)
Non-invasive investigations	6 (33.3)
Pain	5 (27.8)
Other	3 (16.7)
Budget	2 (11.1)

These results pertain to the 18 CHIM studies for which payment factor information was provided by CHIM experts.

CHIM, Controlled Human Infection Model.

## Empirical discussion

Our empirical research found that both the public and CHIM experts believe CHIM participation deserves substantial monetary compensation. Furthermore, they generally believe that CHIMs with a higher level and severity of risk ought to require higher payments. Although, the public more strongly support this claim, whereas our CHIM experts were more divided on the topic. Additionally, we found that currently there is variation in both how payment is determined, and the level of payment offered among CHIMs (see key findings summarised in Box 1).

Box 1 **Key Points**

- CHIM participants are currently paid variable amounts.
- A representative sample of the UK Public believe CHIM participants should be paid approximately triple the minimum wage in the UK and should be paid for the risk they endure through participation.
- CHIM experts believe CHIM participants should be paid more than double the UK minimum wage but are divided on payment for risk.
- The Payment for Risk Model allows risk and pain to be accounted for in CHIM participant payment.
- The payment for risk could be calculated from how risk is calculated in other areas of employment or through using estimates of the value of statistical life.

### Payment attitudes

Overall, the public was more risk averse when it came to allowing participation in CHIM. Their mean WTAP levels were consistently lower than those of the experts over every CHIM, regardless of risk levels. Similarly, the public indicated higher payment than the experts over all levels of risk. The difference noted between the two groups could be a product of how comfortable each group is with the notion of CHIM research. Experts are probably more likely to have a comprehensive understanding of the nature and risk of CHIM research. The public’s responses might instead indicate some trepidation about CHIMs and could suggest a belief that participation is burdensome, risky and even undesirable; hence, it both deserves and requires high payments to fairly compensate participants and to attract enough participants in the first place. On average, both the general public and experts believed that CHIM participants should be paid considerably more than the minimum wage in the UK. Four experts believed their CHIM is currently paying too little for participation, and mean required payment for even the lowest risk level of hypothetical CHIM scenarios was higher than the mean actual payment of the 25 CHIMs we received payment data from. This may indicate that both the public and CHIM experts believe CHIM participants should be paid more than they currently are and provides some support for the concerns that some CHIM participants are currently being underpaid.

The public strongly support the payment for risk in CHIM as both the type of risk and level of risk were observed as main effects in our linear models of the public responses. Furthermore, the public ranked risk as the most important factor to pay for and they indicated a strong level of agreement with statements and payment models involving payment for risk as well as strongly disagreeing with those that did not. This indicates that the public believes it is ethically justified to pay for risk and that they may even consider it as a necessary condition for the fair compensation of CHIM participants. Experts were more divided on the matter as indicated by the type of risk being a significant main effect on our linear models but the level of risk not being significant. Furthermore, many responses ranked risk as the most important payment factor, but many others indicated that it should not be considered in payment at all. We have identified three main reasons as to why some experts may have disagreed with the payment for risk. First, experts may be concerned that if paying for risk is condoned by guidelines and becomes commonplace, they will have to increase their payment to participants that they may not be able to afford. Second, experts may be merely providing answers which align with many of the best practice research guidelines because they believe they ought to do so. Finally, experts may be genuinely concerned that paying for risk could be coercive and/or an undue inducement to CHIM participants. We argue that, out of these three reasons, only the last could render the allowance of higher payment and the payment for risk in CHIM unethical and hence these concerns will be addressed in the next section of this paper. The public did indicate some level of agreement that payment could be coercive and/or an undue inducement. However, these concerns did not prevent them from overwhelmingly supporting the payment for risk and did not lead them to wanting to limit payment to CHIM participants. This indicates that they believe these concerns, although present, are not serious enough to warrant preventative action.

### Payment practices

Our survey findings emphasised that CHIMs are not a homogeneous set of studies and so it is difficult to compare payment between them, yet they share some similarities. In all the studies surveyed, participants were required to be introduced to a pathogen and in most cases, infection with this pathogen was known to carry risks of complications. Furthermore, many participants in these CHIMs were required to endure clinical symptoms and research investigations. Overall, our survey showed being a CHIM participant is quite a burdensome task that puts participants at risk, disrupts a participant’s regular lifestyle and causes them pain, although to varying degrees. Unsurprisingly, we found that there was much variation in the payment offered for each CHIM. However, it seems this variation cannot be fully explained by accounting for the differences in study design, as shown by the stark difference in payment for similar studies involving the same pathogens. We believe that the consideration of different payment factors, which is likely resulting from the lack of a standardised payment framework, is more likely to explain the difference in payment. Interestingly, a substantial number of CHIMs reported to take risk into account in their payment despite many payment guidelines not endorsing this, showing a clear gap between current payment practices and guidelines. This disregard of current guidelines could be indicative of their impracticality and outdatedness.

## Ethical analysis

We will now consider the validity of the main ethical arguments against providing substantial payments and paying for risk in medical research, namely the concerns of coercion and undue inducement. We will argue that these concerns are overstated in current guidelines and risk resulting in unfair underpayment and in some cases exploitation of research participants. Furthermore, we will argue that the claim that these concerns hold more weight in the context of CHIM research is incorrect and that the benefits of paying for the risk in CHIM far outweigh the potential harms.^2^

Finally, we will propose an ethical framework that can be used to determine the ethically sound payment of CHIM participants.

Coercion undermines the condition that informed consent must be voluntary; this is why it invalidates consent and constitutes a serious ethical and legal concern in medical research. According to Alan Wertheimer’s two-pronged test, a coercive proposal must: (A) propose to leave the subject in a worse off position than they would otherwise be in and (B) the subject must have no reasonable alternative other than to comply with the proposal.^39^ In light of this account, it is difficult to see how payment in research could ever be considered coercive as: (A) payment will provide an option that leaves a participant in one way better off and (B) the offer of payment alone will not remove the reasonable alternative of declining participation. In line with this logic, many bioethicists argue that offers of payment, and in fact any offer, cannot coerce, as it leaves a subject better off by expanding their choices.^17 23 40^ Instead, an offer might be exploitative if it takes advantage of injustice or disadvantage unfairly. However, this would be the case if too little is offered for risk, rather than too much. It is exploitative in employment to insufficiently pay for risky labour.

Some of those who have argued that payment offers can be coercive have described situations in which the person giving the offer is somehow directly responsible for the subject’s lack of a reasonable alternative, but this is extremely unlikely to occur in the context of payment for medical research.^41 42^ Despite this, the concerns of ‘coercive payment’ in medical research continue to be widespread. Largent *et al* 43 argue that this is due to ‘conceptual confusion’ and that some people use the term coercion with a different, more liberal definition when referring to payment in medical research payments compared with other scenarios. Recently, Millum and Garnett^44^ proposed a new account of coercion that they believe more accurately describes what is meant by the concerns of coercive payment offers. This account of coercion describes participants who are ‘subjected to a foreign will’ by an offer of payment for research. However, they argue ‘coercion by subjection’ does not invalidate consent and is not necessarily always wrongful. The claim that payment in medical research is ethically worrisome on the account of coercion is misguided. We argue that the offer of payment can be seen as inherently *non-coercive* for the reason that it is expanding a subject’s range of reasonable choices by providing them with the option of receiving fair compensation for the wide range of risks and discomforts that comprise their contribution to medical research. Ethical concerns surrounding the justification for payment in this field may therefore be better understood by either ‘coercion as subjection’ or by undue inducement.

An inducement can be undue if it distorts the recipient’s judgement; that is, it can make a person wrongly assess the risks and benefits of a situation and willingly enter into an agreement that is not in their own best interests.^40^ In theory, this seems like a more reasonable concern; we know that humans are fallible and so the prospect of an offer of money distorting a person’s judgement does not seem outrageous. However, Emanuel^16^ argues that undue inducement is a far ‘too common charge’ in biomedical research that is ‘almost always inaccurate and incorrect’ as regardless of the amount of money offered, payment should never pose undue inducement in medical research because, in theory, the existence of IRBs ought to ensure that no research posing risk of serious harm is ever approved. If it is reasonable to take part in research for no money, or a small amount of money, then it is reasonable to take part in the same research for larger amounts just because the risk is reasonable. Whether IRBs do adequately prevent unacceptably risky research from occurring is another question, although at least in the case of CHIM research, the low rate of serious adverse events seems to indicate that IRBs are somewhat fulfilling their role in that regard.

Other ethicists also argue that the concept of undue inducement in medical research is overly paternalistic as it disallows adults the opportunity to weigh risks and benefits of participation themselves.^45^ It seems that this argument has merit when we consider that adults are not disallowed from undertaking risky professions for money on the account of undue inducement. So why is it different when it comes to participating in medical research? Largent argues the explanation for this discrepancy is the flawed yet widely held belief of research exceptionalism, that is, the concept that ‘research is meaningfully different than other areas of life in which we accept burdens, discomforts and risk’.^17^ Research exceptionalism has led to the strict regulation of research we see today. However, whether it is justified is highly contentious, and it has been strongly disputed that the concern of undue inducement is a valid justification for treating payment in research any different to payment in another job with similar risks, because theoretically undue inducement should pose a risk in both.^17 46^ Furthermore, there is a lack of empirical evidence to show that undue inducement translates from theory into practice, with many studies failing to show that offers of money for medical research distort judgement or risk perception. Instead some studies found that high payments prompted participants to perform more vigilant reviews of the risk information.^47–50^ Ultimately, it may be possible for money to unduly induce a research participant but it is far from clear that this is more likely to occur in CHIM research and that the mere the possibility of undue inducement should warrant the blanket limitation of payment in CHIMs.

Undue inducement has previously been described as a ‘particularly sensitive issue’^15^ in the context of payment for CHIM research and payment in CHIM has attracted a disproportionate amount of media coverage compared with other research that may indicate some support for the notion that payment in CHIM warrants extra caution. We do not believe there is any justification for the claim that undue inducement is more of a concern in CHIM research.

This belief is likely based on the view that CHIM research involves: (A) higher levels of risk and (B) higher levels of payment compared with other research. Furthermore, there may be concern regarding the growing amount of CHIMs being performed in LMICs where participants are more likely to be more economically vulnerable. First, the concern that CHIMs involve higher risk than other research is based on the perception of risks and has no real factual grounding. As previously mentioned, there have only been four serious adverse events reported in modern day CHIMs, a rate comparable with phase I clinical trials. CHIMs may actually attract more vigilant IRB review due to their ‘high risk perception’, which is likely to have led to the large focus on risk minimisation and influenced the high level of inpatient research that allows participants to be more closely monitored than they may be in other research. Second, as we have shown through our payment practices data, CHIM research does not offer considerably high sums of money for participation but in fact offer sums that are far less than what the public and CHIM experts believe should be paid for this work. Therefore, at their current level of payment, we believe participants are at a higher risk of being exploited, not unduly induced. Furthermore, the fact that the goal of CHIM research (to fight infectious disease) is likely to be jointly shared between participants and researchers actually makes it less likely for CHIM participants to be unduly induced into participation by monetary incentives.

A major concern regarding the undertaking of medical research in LMICs is that the benefits of the research will not be adequately reaped by those taking on the burdens.^51^ Take for example testing pharmaceuticals for non-endemic diseases in LMICs and then marketing them solely in HICs. It has been argued that if money is the sole motivator for research participation or if research lacks a shared goal between participants and researchers, it is more likely to ‘coerce as subjection’ or unduly induce participants.^44 52^ However, CHIM research aims to accelerate vaccine production that could lead to a huge decrease in the mortality and morbidity of infectious disease that many CHIM participants, especially in LMICs, have witnessed firsthand in their own communities. Hence, it is unsurprising that CHIM participants in Kenya reported to be motivated by this desire to fight infectious disease as well as compensation.^53^ Similarly, studies looking at motivations of CHIM participants in the UK, which still experience infectious diseases although at lower rates than LMICs, found that the ‘desire to contribute to the progression of medicine’ was the biggest motivator for CHIM participation, even above monetary compensation.^54^ The COVID-19 pandemic has been a cruel reminder that infectious diseases can cause immense economic, social and health impacts from which no country is immune. Hence, it would be reasonable to assume that many, if not all, of those signing up to partake in the COVID-19 CHIM are strongly motivated to find a vaccine that will save lives and allow society to go back to normal, over and above any financial compensation they will receive. The existence of strong motivations for CHIM participation, that go far beyond just the payment, adds another layer of protection against the already unlikely concern that payment could unduly induce participants.^3^

Anomaly and Savulescu^18^ have previously identified numerous benefits of allowing increased payment and the payment for risk in CHIM research.^18^ Most importantly, participants will be at less risk of being exploited. Exploitation occurs when one party takes unfair advantage of another. It is important to note that exploitation can still occur even when both parties benefit from the transaction.^55^ What is bad about exploitation is that the exploitee, even though they may gain, is forced to take an offer with an unfair balance of benefits and risks.^24^ There are two ways to prevent this: correct background injustice (so the exploitee will not choose the offer) or ensure a better balance of benefits and risk. In financial transactions, this may be done by setting a ‘fair price’ for the transaction. However, deciding what constitutes a non-exploitative, fair price for a transaction is far from simple. One way to do so, as proposed by Wertheimer,^56^ is to consider a ‘hypothetical market price’ or ‘the price that would be generated by a competitive market’ for a certain good or service. However, many societies have put in place minimum wage requirements that also need to be satisfied—regardless of what a ‘hypothetical market price’ may be—in recognition of the weak bargaining power that most workers have in comparison with employers, and potential CHIM participants’ bargaining power compared with that of researchers is similarly weak. It is not so simple to use the ‘hypothetical market price’ method to determine a non-exploitative price for CHIM participation because payment is currently not dictated by supply and demand, but it is strictly limited and regulated. Instead, we can reasonably say that a fair minimum price would be one that satisfies the minimum wage of the country in which the study takes place and, as we will argue later, also gives adequate monetary compensation for the risk involved. Our empirical data showed that some CHIMs are paying as little as £1/hour or no money at all. Regardless of whether you use a ‘hypothetical market price’, or the fair minimum price we have suggested above, to settle on a non-exploitative price, a price that low seems like it is at high risk of being exploitative. Researchers and pharmaceutical companies have the potential to make a lot of money from the sale of a vaccine that is approved as a consequence of CHIM data, so it is only fair that the participants, who allow CHIMs to occur, have the opportunity to share in this potential revenue. We believe that current payment guidelines that highlight the concerns of undue inducement and coercion, without paying due attention to the genuine risk of exploitation, may be allowing researchers to defend the gross underpayment of their participants. Furthermore, higher payments will allow the fair compensation for the risk, pain and burdens CHIM participants have to endure throughout a study.^4^ Monetary inducements to take risk are considered ethical in all other areas of employment, and we believe that they are equally ethically justified in medical research as long as participants are well informed of the risk and that the risks have been approved by an IRB. Allowing the payment for risk in CHIMs should in no way alter the level of risk deemed acceptable in this research.

Importantly, there is an ethical obligation for CHIMs to have in place insurance or other pathways that allow for the compensation for any actual harm that occurs as a result of a study, like is often required for other employment. However, from what we said, this alone is not a fair substitute for calculating risk into the payment for a CHIM, as has been previously suggested.^11^ The availability of compensation for actual harm and the upfront payment for potential risk serve two separate purposes. Compensation for harm can be seen as a safety net that ensures participants are not left worse off due to participation in a CHIM. Instead, the payment for risk can be seen as payment for a participant’s willingness to accept risk. Although it may be justified to only provide one, offering both gives the highest level of protection to participants and is the fairest option. Offering only compensation fails to give any benefit to participants who accept risk that does not eventuate in harm. It also fails to recognise that money may not always adequately compensate for all physical harms, especially in the extreme case that death occurs. Without knowing whether all CHIMs offer comprehensive insurance or compensation processes, allowing for the upfront payment for risk will allow some level of protection for participants of studies that may not have adequate harm compensation available. Furthermore, upfront risk payment can act as an inducement and can accelerate recruitment, whereas only offering compensation for harm cannot. This means that in situations where time means lives, such as in the COVID-19 pandemic we are currently witnessing, we do not have to delay studies in order to wait for adequate participants. In other occupations involving risk, such as construction work, there is both payment for risk and compensation for injury. In some jurisdictions, compensation is only available if there is negligence. In others, no-fault compensation exists. We believe payment for risk and no-fault compensation offer the fairest deal for those taking on risky jobs, especially when those jobs are of direct service to society.

We find that the numerous benefits far outweigh the harms of allowing higher payment for CHIM participants. As argued above, payment does not coerce participants and although undue inducement may pose a real concern, it is unlikely to occur, particularly in CHIM research where many participants are often motivated by a desire to contribute to scientific advancement. Furthermore, there is no clear reason as to why the mere possibility of undue inducement should deem all high payment in CHIM ethically unjustifiable. Even so, we support the adoption of robust study design to further minimise the risk. This could include the employment of thorough informed consent processes, which involve the exploration of participants’ motivations and the examination of their understanding of the risks and burdens involved in a study,^2 57^ as well as the use of physiological testing in CHIM participation screening to minimise the potential of payments prompting lying or omission of information that may exclude participants from a CHIM.^11 17^

## An ethical payment framework for CHIM: the payment for risk model

A standardised ethical payment framework for CHIM participants stands to advantage research investigators, participants and the CHIM field as a whole. Investigators would be able to conduct CHIM research with more confidence as their fears of unjust payments risking the exploitation would be diminished. Participants would be more comfortable in consenting to participation with the knowledge that the payment offered fairly compensates them for their involvement. If widely used, a payment framework would strengthen the ethical grounding of CHIM research by establishing a strong sense of justice and fairness when it comes to payment of participants. Justice in the sense that participants are adequately compensated for the risks and burdens they endure and fairness in the sense that participants are rewarded similarly for their involvement in similar studies. Additionally, a standardised framework is likely to decrease competition across models, as payment would be determined using the same principles.

There are specific benefits of employing a standardised payment framework that is informed by both public attitudes as well as ethical arguments. From a practical standpoint, this kind of payment framework is much more likely to be supported by and appeal to the public, as it takes into account their own preferences. Additionally, the transparency of such a framework could work to allay public concerns surrounding payments in CHIM and could help to build public trust in this area of research. From an ethical standpoint, taking into consideration the coherence established between attitudes and normative claims when constructing a standardised payment model can be seen to add some weight to its justification.^26^ A standardised payment system would not be without some disadvantages, mainly it would decrease researchers’ freedom and flexibility to determine their own payments. This seems like a small price to pay for the numerous benefits we have outlined.

We believe the Payment for Risk Model is the best payment model to determine the ethically justifiable payment of CHIM participants. Informed by our novel research findings, this payment model builds on previously described models by allowing for the consideration of risk and pain and is specifically applicable to CHIM research.^52^

### Time: the base rate

The time commitment asked of the participant is the initial factor that is considered in the Payment for Risk Model. We believe payment by an hourly basis, as opposed to a daily or total lump sum of payment, is the easiest and most transparent method for the payment of CHIM participants. It also allows for the calculation of different payment for separate participant groups and easily allows for payment to be prorated. Prorating payment throughout a study is of critical importance so that participants can chose to withdraw consent at any point without the fear losing out financially.^58^ We believe that the base hourly payment rate should be equivalent to that received by an unskilled labourer in the country in which the CHIM is located, as suggested by Dickert and Grady.^52^ However, we argue that this payment alone is not sufficient to deliver ethical payment to CHIM participants. Instead this ‘base rate’ should form the foundation that investigators can then augment. The onus is on researchers to ensure that the base hourly rate adopted satisfies the minimum wage conditions in the country in which the study will occur and is not at risk of being exploitative. If a formal minimum wage does not exist, researchers should enquire into what unskilled labourers are often paid in the country. However, they should be wary that it is likely that some unskilled labourers are underpaid or exploited. Hence, there should be a preference for selecting a higher base rate to minimise this risk.

### Augmentation for risk and pain

Dickert and Grady^52^ endorsed the augmentation of the wage payment model for ‘uncomfortable or burdensome procedures’, however, not for the risk involved in a study. We believe this vague directive is neither clear nor comprehensive enough to lead to the just compensation of CHIM participants. We argue that on top of the time CHIM participants contribute to a study, they should be paid for both the pain they are required to experience and the risk they accept as a result of undergoing procedures or infection. This is exactly what the Payment for Risk Model does.

Unlike in most other jobs, pain and other symptoms are an unpreventable aspect of CHIM participation. We argue that this warrants extra payment due to the significant unforeseen impacts it can have on participants’ mental and physical well-being. Research into the attitudes of CHIM participants has found that the physical requirements of participation can be ‘more significant or more painful than expected’.^59^ This may indicate that more effort is needed to both accurately describe the expected level of pain in a CHIM and to provide adequate compensation for the pain. However, the elusive and subjective nature of pain makes it extremely difficult to accurately quantify, describe and hence determine payment for.

We have previously argued that risk ought to be compensated in CHIM payment. Although in order to do so, an attempt must be made to quantify it. A task that, similar to the evaluation of pain, proves to be very difficult. Evaluating risk is dependent on the availability of high-quality empirical data on the risks and complications of infection and research investigations. Fortunately, there are data describing the complications of commonly performed research investigations such as bronchoscopy^60^ and also for common pathogens used in CHIMs such as influenza and *Plasmodium falciparum*.^61 62^ However, directly using data about investigations performed on patients or community required infections may result in the overestimation of the risks involved in CHIMs that involve healthy participants and employ many risk minimisation strategies.

Despite the challenges of evaluating the risks and the pain involved in a CHIM, it remains critically important that researchers strive to describe these factors for a number of reasons. Primarily, accurate risk and pain information must be portrayed to IRBs so it can be considered in the study approval process. Secondarily, it is crucial this information is available to participants so valid informed consent can be obtained. Finally, it can be used to inform participant payment as we believe it should in the Payment for Risk Model. So, how might we assign a monetary value to the risk or pain involved in a CHIM? To help answer this question, we can examine how money is assigned to these factors in other areas of employment.

American government employees are entitled to ‘hazard pay’ for work that involves ‘extreme physical discomfort and distress’ or work ‘under circumstances in which an accident could result in serious injury or death’.^63^ The ‘hazard pay differential’ for a particular work duty is the percentage of the basic daily wage (ranging from 4% to 25%) that is awarded as additional compensation to those undertaking that duty. Similarly, Australian award pay rates for industries such as building and construction offer a long list of special hourly rate allowances for work involving higher risk and/or physical discomfort.^64^ For example, the ‘acid work allowance’ entitles a worker to an additional $1.93 per hour of work, whereas the ‘hot work allowance – exceeding 54 degrees’ entitles workers to $0.91 per hour. How these amounts were determined is not detailed, so we cannot be sure whether they are arbitrary or a product of careful economic risk calculation. Nonetheless, the notion of forming a risk/pain hierarchy could be easily adopted to rank research investigations and symptoms experienced in a CHIM, prior to assigning hourly award rates or a one-off additional payment for them. Furthermore, the compensation given here may serve as useful reference points for conditions that may require a similar level of discomfort. Additionally, adopting hourly award rates to compensate for symptoms experienced in CHIMs may allow fairer payment for participants who have prolonged or more severe symptoms.

Another method that could be used to assign money to risk is to consider the value of statistical life (VSL), a value that describes ‘the local trade-off rate between fatality risk and money’.^65^ Many countries use estimated VSLs to measure and compare health policy surrounding safety enhancement. It is also a measure of the amount one is willing to pay for an increase in safety, or the amount of money that is needed for one to accept riskier situations. Hence, considering the VSL of the country a CHIM is being performed in could be a useful standard tool to determine the amount of money that we should pay CHIM participants for certain risks. Take for example a hypothetical CHIM to be undertaken in the USA, for which the risk of death as a result of participation is estimated to be 1 in 100 000. Considering the US VSL estimate of 9.6 million, a value used by the Department of Health and Human Services,^66^ and the mortality risk, we can calculate the product of the two giving us an additional risk payment of $96 for the CHIM. An obvious limitation of using the VSL to calculate risk money is that it is only accounting for the risk of death and not of any other complication. To overcome this, the US Department of Transport estimates the value of statistical injury as a fraction of the VSL depending on severity of injury sustained.^67^ A similar calculation could be made to account for adverse events in CHIMs. Alternatively, the product of the likelihood of a complication occurring and the cost of treating the complication (eg, the cost of hospitalisation and treatment) could be used to ascertain the additional risk payment.

We believe either of these two practical methods could be employed to calculate the additional amount of pain and risk compensation a CHIM participant should be given when using the Payment for Risk Model. However, as previously argued, augmented pay for pain and risk should never replace the availability of compensation for any actual harm that may eventuate as a result of participation in a CHIM. We see it as an ethical obligation that research studies have insurance or other means to provide support to participants in the case that actual harm occurs.

## Conclusion

CHIMs are an extremely promising facet of infectious disease research. They even have the potential to help develop a vaccine to COVID-19 and hence to halt the spread of this immensely destructive pandemic. However, in supporting the growth of CHIM research, we must ensure it is being performed in an ethically sound manner and, in particular, that CHIM participants are being fairly compensated for the burdens and risks that they endure. Our empirical research has offered the novel addition of a snapshot of current payment practices in CHIMs, as well as the attitudes of a sample of the UK public and CHIM experts. We found that the current payment in CHIMs is variable and in some cases could be risking the exploitation of CHIM participants. Furthermore, we have found that the UK public strongly believes that the just payment of CHIM participants should involve the compensation for risk, whereas CHIM experts were divided on the issue. Our study findings are limited by the relatively small sample size of CHIM experts we assessed and how our public sample consisted only of UK citizens. Future research could assess these attitudes among a more diverse group. The attitudes of citizens of LMICs in which CHIM research occurs could be of particular interest. Another limitation of our study may be that our survey did not directly assess the attitudes towards our specific payment model. However, despite this, we found that there was strong public support for the key elements included in our model such as paying explicitly for risk and pain.

We considered our empirical results in light of the current normative arguments surrounding the payment of risk and offering high payment in medical research. In order to avoid exploitation, participants must be paid for risk. We argue that the concerns of the payment for risk being coercive or an undue inducement are either incorrect (in the case of coercion) or wrongly over emphasised in our current research guidelines. We believe that CHIM participants deserve to be paid for the pain and risk they endure as they would be if they were working in another risky or uncomfortable job. We have proposed the Payment for Risk Model as a practical and ethical payment model that could be adopted to determine the fair payment of CHIM participants. Although this model has been created specifically in the context of CHIM research, it has the potential to inform payment in other areas of medical research.

10.1136/medethics-2020-106438.supp1 Supplementary data

## Footnotes

Our survey respondents were alerted to the fact that some of the levels of risk employed in our hypothetical scenarios are not reflective of those involved in real CHIMs.

We refer readers to Anomaly and Savulescu’s 2019 paper which has previously set out many of the arguments we will make for allowing higher payments in CHIM.

We would like to thank a reviewer for drawing our attention to the concern of altruistic motivations being exploited. This may be the case in some situations, particularly in those where no payment is offered, but we believe it is very unlikely to occur in CHIM participation. If participants are fully aware of the risks and requirements of participation and give valid informed consent to partake so they fulfil their altruistic desires, then exploitation seems improbable, especially when adequate payment is offered, which can always be declined or donated to charity. If the risk of exploiting altruistic motivations is real, introducing fair payment would prevent, rather than increase this risk.

We would like to acknowledge a reviewer for their comment highlighting that allowing higher payments in CHIM could increase the risk that the researchers providing payments are being exploited by participants. Although this could be possible, the weak bargaining power participants have over researchers makes it extremely unlikely to eventuate in reality.
