# Supplementary material for: Payment in challenge studies: ethics, attitudes and a new payment for risk model
Source: J Med Ethics. 2020 Sep 25;46(12):815–26. doi: 10.1136/medethics-2020-106438 (PMC7719900; doi:10.1136/medethics-2020-106438)
Supplement: Supplementary data [file medethics-2020-106438supp001.pdf]

Supplementary Document: Payment in Challenge Studies: Ethics, Attitudes and a New Payment for Risk Model

Contents:

1. Detailed Explanation of Data Analysis
2. Detailed Description of Additional Payment Attitudes Questions – Public Only
3. Reporting of Additional Results

---

1. Detailed Explanation of Data Analysis

All statistical analyses were performed using the R statistical computing and graphics program. Payment practices information provided in currencies other than GBP were converted to GBP using mean exchange rates over the past year (where available) or exchange rates at the time of survey distribution. Demographic data from our two sample groups were compared using t tests and Chi Square tests. We performed mixed effect linear regression models to explore respondents' willingness to allow participation (WTAP) values and required payment levels across the different levels of risk, the different risk categories, and to compare the two respondent groups. These models allowed us to account for the nested structure of the data and were implemented in *R* using the *lme4* package[1]. Besides subject-level random intercepts, we defined *level of risk*, *type of risk*, and *respondent group* as fixed effects in our main models. We also ran additional models with fixed effects that controlled for covariates such as gender, age, income and baseline risk preferences (risk preferences only in public data).

---

2. Detailed Description of Additional Payment Attitudes Questions – Public Only

Our public respondents answered additional vignette blocks and some further attitudinal questions. The first vignette section described two real and ongoing CHIMs, the information for which were provided to us by the studies' investigators. These questions allowed us to compare respondents' attitudes regarding payment to a small snapshot of real practice. The second block of vignettes explored respondents' attitudes towards the concerns that offers of payment could be coercive or an undue inducement. The independent variable in these

vignettes was the potential participant's "level of need" for the money offered for participation as indicated by a description of their social situation. Respondents were asked to indicate their WTAP level for each scenario. The further attitudinal questions asked respondents to indicate their level of agreement with some payment models and extra statements regarding payment. The five different models of payment presented to respondents were based on those proposed by Dickert and Grady.[2] To avoid misinterpretation, we did not use the terms 'coercion' and 'undue inducement' in statements. Instead, we described the intended effect of payment on the participant to assess respondents' attitudes towards these concerns. To avoid order effect, the order of presentation of these questions was randomised.[3]

---

3. Reporting of Additional Results

Respondent Demographics: There were some statistically significant differences between the demographic characteristics of our two respondent groups (table 1). More specifically, experts were more likely to have attained a higher level of education and to earn a higher income when compared to public respondents.

Real CHIM Scenarios: For both the CHIM scenarios, the mean payment indicated by public respondents was significantly higher than the actual payment provided to CHIM participants in the studies (table 2).

**Characteristic**		**UK Public** *N = 264*	**CHIM Experts** *N = 36*	**Statistical Comparison**
Age (Mean (SD), range)		44.0 (15.0), 18-76	48.1 +/-11.3, 29-70	t(53.4) = -1.97, p > 0.05
Gender	Male	121 (45.8%)	20 (55.6%)	χ^2 (2) = 1.40, p > 0.1
	Female	141 (53.4%)	16 (44.4%)	
	Other	2 (0.8%)	0	
Highest Education Level	Less than high school	1 (0.4%)	0	χ^2 (5) = 78.63, p < 0.001
	High school	43 (16.3%)	0	
	Some college	63 (23.9%)	0	
	2 year degree	21 (8.0%)	0	
	4 year degree	82 (31.1%)	3 (8.3%)	
	Postgraduate degree	54 (20.5%)	33 (91.7%)	
Income (GBP/USD*)	<5000	22 (8.3%)	1 (2.8%)	χ^2 (9) = 70.86, p < 0.001
	5000 – 10000	28 (10.6%)	0	
	10000 – 15000	29 (11.0%)	0	
	15000 – 25000	48 (18.2%)	2 (5.6%)	
	25000 – 35000	41 (15.5%)	3 (8.3%)	
	35000 – 50000	44 (16.7%)	7 (19.4%)	
	50000 – 65000	25 (9.5%)	5 (13.9%)	
	65000 – 80000	17 (6.4%)	3 (8.3%)	
	80000 – 100000	6 (2.3%)	6 (16.7%)	
	>100000	4 (1.5%)	9 (25%)	

Table 1: Demographic characteristics
Continuous data were compared using t tests, discrete data were compared using chi square tests.
*Due to an error the income question was presented to CHIM experts in GBP but to the Public respondents in USD.

CHIM Scenario	Required Payment (GBP/hr) *Mean (SD)*	Actual Payment (GBP/hr)	T Test, p value
Real CHIM 1	18.17 (9.07)	6.96	t(263) = 20.08, p < 0.001
Real CHIM 2	26.44 (10.26)	20	t(263) = 10.20, p < 0.001

Table 2: Real CHIM Scenarios
Respondents were asked to indicate the required payment for participation in £/hr on a slider scale from £0 to >40 for each real CHIM of which they were given a description. The mean required payment as indicated by public respondents were compared to the actual payment (as given to us by the study investigators) by using t tests.

References:

1. Bates D, Mächler M, Bolker B, et al. Fitting Linear Mixed-Effects Models Usinglme4. *Journal of Statistical Software* 2015;67(1) doi: 10.18637/jss.v067.i01
2. Dickert N, Grady C. What's the price of a research subject? Approaches to payment for research participation. *The New England Journal of Medicine* 1999;341
3. Strack F. “Order Effects” in Survey Research: Activation and Information Functions of Preceding Questions. In: Schwarz N, Sudman S, eds. Context Effects in Social and Psychological Research. New York: Springer 1992.
